# Ophiostomatoid fungi associated with pines infected by *Bursaphelenchusxylophilus* and *Monochamusalternatus* in China, including three new species

**DOI:** 10.3897/mycokeys.39.27014

**Published:** 2018-09-04

**Authors:** HuiMin Wang, YingYing Lun, Quan Lu, HuiXiang Liu, Cony Decock, XingYao Zhang

**Affiliations:** 1 Key Laboratory of Forest Protection State Forestry Administration, Research Institute of Forest Ecology, Environment and Protection, Chinese Academy of Forestry, Beijing 100091, China; 2 College of Plant Protection of Shandong Agricultural University, Taian 271018, China; 3 Longju Ecological Forest Farm, Dongying 257085, China; 4 Mycothèque de l’Université Catholique de Louvain (MUCL), Earth and Life Institute, Microbiology, B-1348 Louvain-la-Neuve, Belgium

**Keywords:** *
Ophiostoma
*, taxonomy, *
Sporothrix
*, *Ophiostomaminus* complex, *Ophiostomaips* complex

## Abstract

The activity of the pine wood nematode *Bursaphelenchusxylophilus* leads to extremely serious economic, ecological and social losses in East Asia. The nematode causes pine wilt disease, which is currently regarded as the most important forest disease in China. The pathogenic nematode feeds on dendrocola fungi to complete its cycle of infection. As the vector of the nematode, the Japanese pine sawyer (*Monochamusalternatus*) also carries dendrocola fungi. Pine woods, infected by *B.xylophilus* and tunnelled by *M.alternatus*, are also inhabited by ophiostomatoid fungi. These fungi are well known for their association with many bark and ambrosia beetles. They can cause sapstain and other serious tree diseases. The aims of our study were to investigate and identify the ophiostomatoid communities associated with the epidemic pine wood nematode and the pine sawyer in *Pinusmassoniana* and *P.thunbergii* forests, which are the main hosts of the pine wood nematode in China. Two hundred and forty strains of ophiostomatoid fungi were isolated from nematode and sawyer–infected trees in the coastal Shandong and Zhejiang Provinces, representing newly and historically infected areas, respectively. Six ophiostomatoid species were identified on the basis of morphological, physiological and molecular data. For the latter, DNA sequences of the internal transcribed spacer (ITS1–5.8S–ITS2) region and partial b-tubulin gene were examined. The ophiostomatoid species included one known species, *Ophiostomaips*, three novel species, viz. *Ophiostomaalbum***sp. nov.**, *Ophiostomamassoniana***sp. nov.** and *Sporothrixzhejiangensis***sp. nov.** and two species whose identities are still uncertain, Ophiostomacf.deltoideosporum and Graphilbumcf.rectangulosporium, due to the paucity of the materials obtained. The ophiostomatoid community was dominated by *O.ips*. This study revealed that a relatively high species diversity of ophiostomatoid fungi are associated with pine infected by *B.xylophilus* and *M.alternatus* in China.

## Introduction

The pathogenic pine wood nematode (PWN) *Bursaphelenchusxylophilus* (Steiner & Buhrer) Nickle (Aphelenchida, Parasitaphelenchidae), presumably native to North America (Steiner and Buhrer 1934, Robbins 1982, Ryss et al. 2005, Zhao et al. 2014), is a mild threat to pine trees in its native area. Nevertheless, this species and the concomitant systematic wilt symptom are responsible for pine tree deaths affecting many trees in eastern Asia, notably in Japan and China (Evans et al. 1996, Mota and Vieira 2008, Mamiya and Shoji 2009, Jung 2010, Futai 2013). Since the first report in China, in Nanjing City in 1982, the disease has spread through more than 300 counties in the provinces of Jiangsu, Zhejiang, Shandong and others, which are currently listed as PWN epidemic areas (State Forestry Administration of the People’s Republic of China 2018). The wilt disease has caused enormous losses not only to the economy and ecology, but also to society, becoming one of the most serious ecological devastation events in Chinese forests.

*Bursaphelenchusxylophilus* infects many species of coniferous trees, mainly from the genus *Pinus* (Yan et al. 2003). *Pinusarmandii*, P.kesiyavar.langbianensis, *P.koraiensis*, *P.massoniana*, *P.tabuliformis*, *P.taiwanensis*, *P.thunbergii* and *P.yunnanensis* are naturally infected by PWN in China (Zhao and Sun 2017). During the infection cycle, the nematode needs vector beetles for dispersal and inoculation into new hosts. The Japanese pine sawyer, *Monochamusalternatus* Hope (Coleoptera, Cerambycidae), is considered to be the primary PWN vector indigenous to Asia. At the initial stage of infection, PWN feeds on epithelial cells of the host pine (Mota and Vieira 2008, Zhao et al. 2008, Futai 2013). Upon tree death, it feeds on the dendrocola fungi to maintain its population and propagate (Suh et al. 2013, Zhao et al. 2013, 2014).

The ophiostomatoid fungi are one of the most common fungal groups inhabiting wood infected by *B.xylophilus*. Further, many ophiostomatoid reproduction structures are detected in the tunnels of *M.alternatus*, suggesting a relationship between the fungi and the occurrence and development of the disease. For instance, *O.ips* has been found in the PWN vector beetles in North America, China and Korea (Wingfield 1987, Suh et al. 2013, Zhao et al. 2014). There is some evidence that the fungi adhere to the body surface of adult *M.alternatus* and thus are transmitted to the twigs of healthy trees (Suh et al. 2013).

The association of PWN with ophiostomatoid fungi and bacteria likely contributes to the nematode’s pathogenicity (Zhao et al. 2013, Zhao and Sun 2017). *Ophiostomaminus* and *Sporothrix* sp. can stimulate the reproduction of PWN and, consequently, the numbers of PWN carried by the emerging beetles (Maehara and Futai 1997, Zhao et al. 2013, Zhao and Sun 2017). Moreover, the fragrant diacetone alcohol released from wood infected by *Sporothrix* sp. 1 can induce *B.xylophilus* to produce greater number of offspring and promotes beetle growth and survival (Zhao et al. 2013).

Thus far, the association with PWN and *Monochamus* spp. has been documented for only five species of ophiostomatoid fungi worldwide (Wingfield 1987, Maehara and Futai 1997, Hyun et al. 2007, Suh et al. 2013, Zhao et al. 2013, Zhao and Sun 2017). Determination of the identities of these species is mainly based on morphology and sequence comparisons of a single DNA locus. Given the diversity of ophiostomatoid fungi associated with other beetles, the serious impact of the nematode and sawyers on wood and the potential importance of these fungi in the disease infection cycle, studies of the diversity and occurrence of the ophiostomatoid fungi involved in the pine wilt disease should be intensified. Such studies will enable understanding of the interaction between the disease system and the fungi, ultimately helping to redress the current situation of the ceaseless outbreaks and rapid expansion of the disease.

The aims of the current study were to investigate and identify the ophiostomatoid mycobiota associated with the nematode and sawyer in the epidemic forests of Shandong and Zhejiang Provinces in eastern China to facilitate the understanding of pine wilt disease infection and prevalence mechanisms. The two coastal provinces, Shandong and Zhejiang, represent new and historic epidemic areas, with *P.thunbergii* and *P.massoniana* as hosts, respectively.

## Materials and methods

### Collection of samples and fungus isolations

Fungi were isolated from 98 samples of *M.alternatus* galleries or pupal chambers in *P.massoniana* and *P.thunbergii* in the Zhejiang and Shandong Provinces (Table 1), in November 2012. All host trees used for sample collection in this study were exhibiting weak or dying symptoms, blue stain and 4–5 instar larvae residing inside after dissecting the stems. The nematodes were also isolated from these galleries and pupal chambers by Behrman funnel. The fungi were isolated on the surface of 2% (w/v) water agar (20 g agar powder in 1000 ml of deionised water) in 9 cm wide Petri dishes and incubated at 25 °C (Seifert et al. 1993, Zhao et al. 2013, Chang et al. 2017). Subsequently, all strains were purified by hyphal tip isolation, using the procedure described by Jacobs and Wingfield (2001) and routinely grown on 2% (w/v) malt extract agar (MEA; 20 g malt extract powder and 20 g agar powder in 1000 ml of deionised water). Representative cultures were deposited in the China Forestry Culture Collection Center (CFCC), culture collection of the Chinese Academy of Forestry (CXY) and part of the Belgian Coordinated Collections of Microorganisms (MUCL), culture collection at Université Catholique de Louvain, Belgium.

**Table 1.** Strains of ophiostomatoid fungi isolated from pines infested by Monochamusalternatus and pine wood nematode in the current study.

Group	Species	Strain No.	Host	Origin (Latitude, Longitude)	Genbank No.		Collector
					ITS	β-tubulin	
A	*Sporothrixzhejiangensis* sp. nov.	MUCL 55181 (CFCC52167, CXY1612)	* Pinus massoniana *	Yuyao, Zhejiang (29°58'10.2"N, 121°05'57.1"E)	KY094069	MH397728	Q. Lu, YY Lun
		MUCL 55182 (CFCC52164, CXY1613)	* P. massoniana *	Yuyao, Zhejiang (29°58'10.2"N, 121°05'57.1"E)	KY094070	MH397729	
		MUCL 55183 (CFCC52165, CXY1614)	* P. massoniana *	Yuyao, Zhejiang (29°58'10.2"N, 121°05'57.1"E)	KY094071	MH397730	
		MUCL 55184 (CFCC52166, CXY1615)	* P. massoniana *	Yuyao, Zhejiang (29°58'10.2"N, 121°05'57.1"E)	KY094072	MH397731	
B	*Ophiostomaalbum* sp. nov.	MUCL 55189 (CFCC52168, CXY1622)	* P. massoniana *	Yuyao, Zhejiang (29°58'10.2"N, 121°05'57.1"E)	KY094073	MH360979	
		MUCL 55190 (CFCC52169, CXY1642)	* P. massoniana *	Yuyao, Zhejiang (29°58'10.2"N, 121°05'57.1"E)	KY094074	MH360980	
		CFCC52170 (CXY1643)	* P. massoniana *	Yuyao, Zhejiang (29°58'10.2"N, 121°05'57.1"E)	KY094075	MH360981	
C	* Ophiostoma ips *	CXY1628	* P. thunbergii *	Changdao, Shandong (37°59'13.5"N, 120°42'18.1"E)	KY593324	MH324804	
		CXY1631	* P. thunbergii *	Zhoushan, Zhejiang (29°52'51.33"N, 122°24'14.13"E)	MH324811	MH324805	
		CXY1635	* P. massoniana *	Yuyao, Zhejiang (29°58'10.2"N, 121°05'57.1"E)	MH324812	MH324808	
		CXY1638	* P. thunbergii *	Fuyang, Zhejiang (30°05'15.1"N, 119°58'55.1"E)	MH324813	MH324809	
		CXY1639	* P. massoniana *	Weihai, Shandong (37°23'23.6"N, 122°32'33.1"E)	MH324814	MH324810	
D	*Ophiostomamassoniana* sp. nov.	MUCL 55179 (CFCC51648, CXY1610)	* P. massoniana *	Fuyang, Zhejiang (30°05'15.1"N, 119°58'55.1"E)	KY094067	MH370810	
		MUCL 55180 (CFCC51649, CXY1611)	* P. massoniana *	Yuyao, Zhejiang (29°59'36.87"N, 121°09'09.90"E)	KY094068	MH370811	
E	Graphilbum cf. rectangulosporium	CXY1623	* P. massoniana *	Yuyao, Zhejiang (29°59'36.87"N, 121°09'09.90"E)	MH324816	–	
F	Ophiostoma cf. deltoideosporum	MUCL 55191 (CXY1640)	* P. thunbergii *	Weihai, Shandong (37°23'23.6"N, 122°32'33.1"E)	MH324815	–	

MUCL: part of the Belgian Coordinated Collections of Microorganisms; CFCC: China Forestry Culture Collection Center; Beijing, China; CXY (Culture Xingyao): culture collection of the Research Institute of Forest Ecology, Environment, and Protection, Chinese Academy of Forestry.

Sequences missing data are indicated by [–].

### Culture and morphological studies

The ophiostomatoid fungal strains were incubated on 2% MEA and 2% potato dextrose agar (PDA; 200 g potato and 20 g dextrose, 20 g agar powder in 1000 ml of deionised water: the dextrose was obtained from American Amresco) in the dark at 25 °C in an incubator. Fungal growth on MEA plates was monitored daily. Hyphal tips of emerging colonies were transferred to fresh MEA plates to purify the fungi. Slides were made to observe the sexual/asexual state structures; these were mounted in lactic acid cotton blue on glass slides and examined under a BX51 OLYMPUS microscope. Fifty measurements were made of each microscopic taxonomically informative structure. The measurements are presented in the form: (minimum–) mean minus standard deviation–mean plus standard deviation (–maximum).

A 5-mm mycelium disc was cut from an actively growing fungal colony using a sterile cork borer and placed at the centre of MEA plates, with the aerial mycelium side in contact with the medium. Three replicate plates were prepared for each strain and were incubated at temperatures ranging from 5–40 °C at five-degree intervals. The colony diameters on each Petri dish were determined along two perpendicular axes every day until the entire dish was covered. The colour descriptions were provided according to Rayner (1970).

### DNA extraction, PCR and sequencing reactions

DNA was extracted from freshly collected mycelia grown in liquid malt medium (20g malt extract in 1000 ml of deionised water) at 25 °C in the dark for 7 d using an Invisorb Spin Plant mini kit (Invitek, Berlin, Germany), following the manufacturer’s instructions. The internal transcribed spacer (ITS) regions and partial β–tubulin (*tub2*) genes were amplified using primer pairs ITS1/ITS4 (White et al. 1990) and Bt2a/Bt2b (Glass and Donaldson 1995), respectively.

PCR reactions were performed in 25 ml volumes (2.5 mM MgCl_2_, 1X PCR buffer, 0.2 mM dNTP, 0.2 mM of each primer and 2.5 U of Taq polymerase). The conditions for ITS and *tub2* PCR amplifications were as described earlier (White et al. 1990, Glass and Donaldson 1995). PCR products were purified using an MSB Spin PCRapace kit (250) (Invitek), following the manufacturer’s instructions.

Sequencing reactions were performed using CEQ DTCS Quick Start KitH (Beckman Coulter, American), following the manufacturer’s instructions, with the same PCR primers as above. Nucleotide sequences were determined using a CEQ 2000 XL capillary automated sequencer (Beckman Coulter).

### Phylogenetic analyses

Contigs were subjected to BLAST searches of the NCBI GenBank database (https://www.ncbi.nlm.nih.gov/); published sequences of closely related species were retrieved. Alignments of the related genes (most up-to-date sequence regions deposited in the GenBank) were conducted online using MAFFT v 7.0 (https://mafft.cbrc.jp/alignment/server/index.html) (Katoh and Standley 2013) and the E-INS-i strategy. Subsequently, the datasets were checked manually by using MEGA v 5.2 (Tamura et al. 2011). Gaps were treated as a fifth base. Phylogenetic analyses were performed using maximum parsimony (MP), as implemented in PAUP* v 4.0b10 (Swofford 2003); Bayesian Inference (BI), as implemented in MrBayes v 3.1.2 (Huelsenbeck and Ronquist 2001); and Maximum Likelihood (ML), using PhyML v 3.0 (Guidon and Gascuel 2003).

The most parsimonious trees generated by MP analyses were identified by heuristic searches with a random addition sequence (1000); max trees were set to 200 and further evaluated by bootstrap analysis, retaining clades compatible with the 50% majority rule in the bootstrap consensus tree. The analysis was based on tree bisection reconnection branch swapping (TBR). The tree length (TL), consistency index (CI), retention index (RI), homoplasy index (HI) and rescaled consistency index (RC) were recorded for each dataset after tree generation.

The general-time-reversible (GTR) model for ML analyses was selected using the Akaike Information Criterion (AIC) in ModelTest v 3.7 (Posada and Crandall 1998). ML runs performed using the CIPRES cluster at the San Diego Supercomputing Center (USA). Node support was estimated from 1000 bootstrap replicates.

For BI analyses, the most appropriate substitution models were also selected using the general-time-reversible model (GRT) with AIC in ModelTest v 3.7. BI was carried out with MrBayes using the Markov Chain Monte Carlo (MCMC) approach with 5,000,000 generations, to estimate posterior probabilities.

## Results

### Fungal isolation and sequence comparison

In total, 240 strains belonging to Ophiostomatales were obtained from PWN-infected galleries and pupal chambers of *M.alternatus*. The strains were sorted into six morphological groups (groups A–F in Table 1), tentatively identified as *Sporothrix*, *Ophiostoma* and *Graphilbum*. After preliminary ITS sequence comparisons of all these strains, 11 strains were clearly disparate to any known species and the remaining 229 strains possessed > 99% similarity with type strain of *O.ips* (GenBank no. AY546704).

### Phylogenetic analyses

ITS and *tub2* sequences were generated for 16 strains and deposited in GenBank (Table 1). The ITS alignment matrix contained 110 sequences (Tables 1 and 2) and 651 characters, including gaps, following the preliminary determination of strain affinities using the BLAST search engine (GenBank). Due to the presence or absence in intron in the *tub2* sequence in the *Sporothrix* and *Ophiostoma* lineage species (Zipfel et al. 2006, de Beer et al. 2016), three separate datasets were built for the *tub2* sequences. These were *Sporothrix*, *Ophiostomaminus* complex and *Ophiostomatenellum* complex datasets (Linnakoski et al. 2010, de Beer et al. 2013, 2016). The *Sporothrix* dataset contained 8 species, 17 sequences and 403 characters, including gaps. The *O.minus* dataset contained 5 species, 17 sequences and 447 characters, including gaps. The *O.tenellum* dataset contained 8 species, 14 sequences and 280 characters, including gaps.

**Table 2.** The information of references sequences used for phylogenetic analyses in this study.

Species	Strain No.	Host/insect	Country	Genbank No.		Reference
				ITS	β-tubulin	
* Sporothrix abietina *	CBS125.89	* Abies vejari *	Mexico	AF484453	KX590755	de Beer et al. 2003
* S. aurorae *	CMW19362	* Pinus eliottii *	South Africa	DQ396796	DQ396800	Francois et al. 2006
* S. bragantina *	CBS 474.91	Soil	Brazil	FN546965	FN547387	Madrid et al. 2010
	CBS 430.92	Soil	Brazil	FN546964	FN547386	Madrid et al. 2010
* S. brasiliensis *	Ss383	* Felis catus *	Brazil	KP890194	FN547387	Araujo et al. 2015
* S. brunneoviolacea *	CBS 124562	Soil	Spain	FN546959	FN547385	Madrid et al. 2010
	CBS 124564	Soil	Spain	FN546958	FN547384	Madrid et al. 2010
* S. dentifunda *	CMW13016	Quercus wood	Hungary	AY495434	AY495445	Aghayeva et al. 2005
	CMW13017	Quercus wood	Poland	AY495435	AY495446	Aghayeva et al. 2005
* S. epigloea *	CBS 573.63	* Tremella fusiformis *	Argentina	KX590817	KX590760	de Beer et al. 2016
* S. eucalyptigena *	CPC 24638	* Eucalyptus marginata *	Western Australia	KR476721	N/A	Crous et al. 2015
* S. gemella *	CMW23057	* Protea caffra *	South Africa	DQ821560	DQ821554	Roets et al. 2008
* S. inflata *	CMW12529	Soil	Canada	AY495428	AY495438	Aghayeva et al. 2005
	CMW12527	wheat-field soil	Germany	AY495426	AY495437	Aghayeva et al. 2005
* S. nebularis *	CMW27319	* Orthotomicus erosus *	Spain	DQ674375	N/A	Romón et al. 1900
	CMW27900	* O. erosus *	Spain	DQ674376	N/A	Romón et al. 1900
* S. pallida *	CBS131.56	* Stemonitis fusca *	Japan	EF127880	EF139110	de Meyer et al. 2008
	CBS150.87	* S. fusca *	Japan	EF127879	EF139109	de Meyer et al. 2008
* S. palmiculminata *	CMW23049	* Protea repens *	South Africa	DQ316191	DQ821543	Francois et al. 2006
* S. phasma *	CMW20676	* P. laurifolia *	South Africa	DQ316219	DQ821541	Francois et al. 2006
* S. proteara *	CMW1103	* P. caffra *	South Africa	DQ316203	DQ316165	Francois et al. 2006
* S. schenckii *	MITS2474	N/A	Mexico	KP132783	N/A	Irinyi et al. 2015
	CBS 938.72	Human	Franch	KP017094	N/A	Irinyi et al. 2015
* S. fusiforis *	CMW9968	* Populus nigra *	Azerbaijan	AY280481	AY280461	Aghayeva et al. 2004
* S. lunata *	CMW10563	* Carpinus betulus *	Austria	AY280485	AY280466	Zhou et al. 2006
* S. narcissi *	CBS138.50	N/A	Canada	AY194510	KX590765	Jacobs et al. 2003
* S. splendens *	CMW872	* Protea repens *	South Africa	DQ316215	DQ316177	Francois et al. 2006
* S. stenoceras *	CMW2524	* Acacia mearnsii *	South Africa	AF484459	AY280473	de Beer et al. 2003
	CBS237.32	pine pulp	Norway	AF484462	N/A	de Beer et al. 2003
* S. thermara *	CMW38930	* Euphorbia ingens *	South Africa	KR051115	KR051103	Ja et al. 2016
	CMW38929	* E. ingens *	South Africa	KR051114	KR051102	Ja et al. 2016
* S. stylites *	CMW14543	Pine utility poles	Australia	EF127883	EF139096	de Meyer et al. 2008
* Ophiostoma adjuncti *	CMW135	* Pinus ponderosa *	USA	AY546696	N/A	Zhou et al. 2004
* O. allantosporum *	CBS185.86	* P. pinaster *	Europe	AY934506	N/A	Villarreal et al. 2005
* O. angusticollis *	Zoq16	N/A	N/A	EU109671	N/A	de Beer et al. 2016
	CBS186.86	* Pinus banksiana *	USA	AY924383	KX590757	Villarreal et al. 2005
* O. bicolor *	CBS492.77	*Piceaglauca*/*Ips* sp.	USA	DQ268604	DQ268635	Massoumi et al. 2007
* O. candidum *	CMW26484	* Eucalyptus cloeziana *	South Africa	HM051409	HM041874	Nkuekam et al. 2012
	CMW26483	* E. cloeziana *	South Africa	HM051408	HM041873	Nkuekam et al. 2012
* O. catonianum *	C1084	* Pyrus *	Italy	AF198243	N/A	Gorton et al. 2004
* O. coronatum *	CBS 497.77	* Pinus pinaster *	Iberian Peninsula	AY924385	KX590758	Villarreal et al. 2005
* O. cupulatum *	C1194	* Pseudotsuga *	USA	AF198230	N/A	Uzunovic et al. 2000
* O. deltoideosporum *	WIN(M)41	N/A	N/A	EU879121	N/A	Mullineux and Hausner 2009
* O. fasciatum *	UM56	* Pseudotsuga menziesii *	CanadaCanada	EU913720	EU913759	Plattner et al. 2009
* O. floccosum *	C01-021	Girdled *Picearubens*	Canada	AY194504	N/A	Jacobs et al. 2003
	C1086	Soil	Sweden	AF198231	N/A	Gorton et al. 2004
* O. fumeum *	CMW26813	* Eucalyptus cloeziana *	South Africa	HM051412	HM041878	Nkuekam et al. 2012
	CMW26818	* E. cloeziana *	South Africa	HM051415	HM041877	Nkuekam et al. 2012
* O. fuscum *	CMW23196	* Picea abies *	Finland	HM031504	HM031563	Linnakoski et al. 2010
* O. himai ulmi *	C1183	* Ulmus *	India	AF198233	N/A	Harrington et al. 2001
	C1306	* Ulmus *	India	AF198234	N/A	Harrington et al. 2001
* O. ips *	CMW7075	N/A	USA	AY546704	N/A	Zhou et al. 2004
	CMW22843	* Orthotomicus erosus *	N/A	DQ539549	N/A	Romón et al. 2007
* O. japonicum *	YCC099	N/A	N/A	GU134169	N/A	Yamaoka et al. 2009
* O. kryptum *	DAOM 229701	*Piceaabies*/*Tetropium* sp.	Austria	AY304436	AY305685	Jacobs and Kirisits 2013
	DAOM 229702	*Larixdecidua*/*T.gabrieli*	Austria	AY304434	AY305686	Jacobs and Kirisits 2013
	K6/3/2	*Piceaabies*/*Tetropium* sp.	Austria	AY304428	AY305687	Jacobs and Kirisits 2013
* O. minus *	PIR 18S	N/A	N/A	AY934509	N/A	Villarreal et al. 2005
	CMW22802	* Dryocoetes autographus *	N/A	DQ539507	N/A	Romón et al. 2005
	RJ-T144	*Tetropium* sp.	Poland	AM943886	N/A	Jankowiak and KolařÍk 2010
	CMW28117	*Piceaabies*/*Tomicusminor*	Russia	HM031497	HM031535	Linnakoski et al. 2010
	AU58.4	* Lodgepole pine *	Canada	AF234834	N/A	Gorton et al. 2004
	DAOM 212686	N/A	Canada	AY304438	AY305690	Jacobs and Kirisits 2013
* O. micans *	CMW:38903	* Picea crassifolia *	China	KU184432	KU184303	Yin et al. 2016
* O. montium *	CMW13221	*Pinusponderosa*/ *Dendroctonusponderosae*	USA	AY546711	N/A	Zhou et al. 2004
	CMW13222	*P.contorta*/*D.ponderosae*	Canada	AY546712	N/A	Zhou et al. 2004
* O. nigrocarpum *	CMW 560	*Abies* sp.	USA	AY280489	AY280479	Aghayeva et al. 2004
	CMW651	* Pseudotsuga menziesii *	USA	AY280490	AY280480	Aghayeva et al. 2004
* O. nitidum *	CMW:38907	* Picea crassifolia *	China	KU184437	KU184308	Yin et al. 2016
* O. novo ulmi *	C1185	* Ulmus *	Russia	AF198235	N/A	Harrington et al. 2001
	C510	* Ulmus *	USA	AF198236	N/A	Harrington et al. 2001
* O. olgensis *	CXY1404	*Larixgmelini*/*Ipssubelongatus*	China	KU551299	KU882938	Wang et al. 2016
	CXY1405	*L.gmelini*/*I.subelongatus*	China	KU551300	KU882939	Wang et al. 2016
	CXY1410	*L.gmelini*/*I.subelongatus*	China	KU551303	KU882942	Wang et al. 2016
* O. pallidulum *	CMW23279	*Pinussylvestris*/*Hylastesbrunneus*	Finland	HM031509	N/A	Linnakoski et al. 2010
	CMW23278	*P.sylvestris*/ *H.brunneus*	Finland	HM031510	HM031566	Linnakoski et al. 2010
* O. piceae *	C1087	N/A	Germany	AF198226	N/A	Uzunovic et al. 2000
	C1246	* Pseudotsuga *	USA	AF198227	N/A	Uzunovic et al. 2000
* O. pseudotsugae *	92-634/302/6	*Pinusmenziesii*/*Dendroctonusfrontalis*	Canada	AY542502	AY548744	Gorton et al. 2004
	D48/3	N/A	Canada	AY542501	AY542511	Gorton et al. 2004
* O. proteasedis *	CMW *28601*	* Protea caffra *	Zambia	EU660449	EU660464	Roets et al. 2009
* O. pulvinisporum *	CMW9022	*Pinuspseudostrobus*/*Dendroctonusmexicanus*	Mexico	AY546714	DQ296100	Zhou et al. 2004
* O. qinghaiense *	CMW:38902	* Picea crassifolia *	China	KU184445	KU184316	Yin et al. 2016
* O. querci *	C970	* Quercus *	United Kingdom	AF198239	N/A	Gorton et al. 2004
	C969	* Quercus *	United Kingdom	AF198238	N/A	Gorton et al. 2004
	C1085	* Fagus *	Germany	AF198237	N/A	Gorton et al. 2004
* O. rostrocoronatum *	CBS434.77	Woodpulp	USA	AY194509	KX590771	Jacobs et al. 2003
* O. saponiodorum *	CMW29497	*Piceaabies*/*Ipstypographus*	Finland	HM031507	HM031571	Linnakoski et al. 2010
	CMW28135	* P. abies *	Russia	HM031508	N/A	Linnakoski et al. 2010
* O. sejunctum *	Ophi 1B	N/A	N/A	AY934520	N/A	Villarreal et al. 2005
	Ophi 1A	N/A	N/A	AY934519	N/A	Villarreal et al. 2005
* O. setosum *	AU160-38	* Pseutotsugae menziesii *	North America	AF128929	N/A	Uzunovic et al. 2000
	CMW12378	*Tsuga* sp.	China	FJ430485	FJ430515	Grobbelaar et al. 2009
* O. tenellum *	CBS189.86	* Pinus banksiana *	USA	AY934523	KX590772	Villarreal et al. 2005
* O. tetropii *	C00-027a	* Tetropium fuscum *	Canada	AY194482	NA	Jacobs et al. 2003
	C00-003	* T. fuscum *	Canada	AY194485	AY305701	Jacobs et al. 2003
* O. ulmi *	C1182	* Ulmus *	Netherlands	AF198232	N/A	Harrington et al. 2001
* Graphilbum crescericum *	CMW 22829	* Hylastes ater *	Spain	DQ539535	N/A	Romón et al. 2007
* Gra. fragrans *	C1224	* Pinus sylvestris *	Sweden	AF198248	N/A	Harrington et al. 2001
* Gra. microcarpum *	YCC612	Japanese larch logs	Japan	GU134170	N/A	Yamaoka et al. 2009
* Gra. rectangulosporium *	MAFF 238951	N/A	Japan	AB242825	N/A	Ohtaka et al. 2006
* Raffaelea canadensis *	CBS 168.66	N/A	N/A	GQ225699	N/A	Kyunghee et al. 2009
* Leptographium lundbergii *	DAOM 64746	N/A	N/A	EU879151	AY534943	Mullineux and Hausner 2009
* L. truncatum *	WIN(M)1435	* Pinus taeda *	South Africa	AY935626	N/A	Hausner et al. 2005

ITS = internal transcribed spacer regions 1 and 2 of the nuclear ribosomal DNA operon, including the 5.8S region; *tub*2 = beta-tubulin;

N/A= represents information that are not available.

CMW = Culture Collection of the Forestry and Agricultural Biotechnology Institute; CBS = The culture collection of Westerdijk Fungal Biodiversity Institute, Utrecht, the Netherlands; MAFF = Ministry of Agriculture, Forestry, and Fisheries, Genetic Resource Centre, Culture Collection of National Institute of Agrobiological Resources, Japan; CXY (Culture Xingyao): Culture collection of the Research Institute of Forest Ecology, Environment and Protection, Chinese Academy of Forestry.

**Figure 1.**
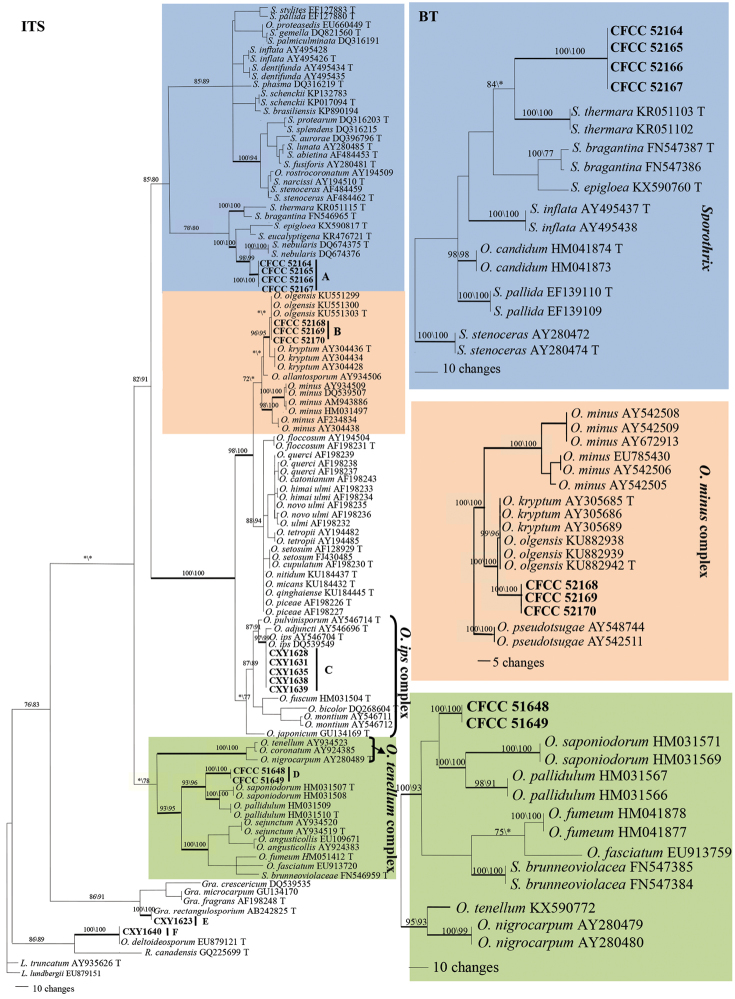
Phylograms of fungal associates of pine infected by PWN and *Monochamusalternatus* in China. The phylograms were generated after MP analysis of the ITS1–5.8S–ITS2 rDNA and partial *tub2* sequences. Novel sequences obtained in the current study are indicated in bold type. MP bootstrap values (10,000 replicates) and ML bootstrap support values (1000 replicates) (normal type) above 70% are indicated at the nodes. Values below 70% are indicated by asterisk (*). Posterior probabilities (above 90%) obtained from BI are indicated by bold lines at the relevant branching points. Scale bar, total nucleotide differences between taxa; ML, maximum likelihood; MP, maximum parsimony; BI, Bayesian inference.

For each phylogenetic tree, MP, ML and BI analyses yielded trees with very similar topologies. Phylograms, generated by the MP analysis, are presented for all the datasets, with nodal support obtained from ML indicated at the nodes (Figure 1). In addition, posterior probabilities (above 90%), obtained from BI, are indicated by bold lines at the relevant branching points. Analyses of the ITS1–5.8S–ITS2 region revealed that the analysed strains formed six distinct clades (Figure 1).

According to the ITS sequence analysis, strains of the morphological group A nested in the *Sporothrix* lineage, as defined by de Beer et al. (2016). They form a well-supported independent clade, closely related to *S.nebularis*, *S.epigloea* and *S.eucalyptigena*. Strains exhibiting morphotypes B, C and D formed three clades in the *Ophiostomas. str* lineage (de Beer and Wingfield 2013). Group B strains nested in the *O.minus* complex, with *O.olgensis* forming a well-supported clade, which closely related to *O.kryptum* (Linnakoski et al. 2010, de Beer and Wingfield 2013, Wang et al. 2016). Group C strains nested within the well-supported *O.ips* clade. Group D strains nested within the *Ophiostoma* lineage and closely related to *O.saponiodorum* and *O.pallidulum*. Finally, strains exhibiting morphotypes E and F nested in the *Graphilbum* and *Raffaeleas. l.* lineages, respectively (de Beer and Wingfield 2013) (TL=821, CI=0.5445, RI=0.8046, HI=0.4555, RC=0.4381 in the MP phylogenetic tree).

Phylogenetic inferences based on *tub2* sequences revealed that clade A, B and D strains formed three well-supported independent clades within the *Sporothrix* and *Ophiostoma* lineages, respectively. Clade C strains nested within the well-supported *O.ips* clade (Suppl. material 1).

Considering morphological differences, strains in groups A, B and D represent three undescribed species of *Sporothrix* or *Ophiostoma*. We concluded that group C strains belong to *O.ips*; group E and F strains clustered together with the well-supported *Graphilbumrectangulosporium* and *O.deltoideosporum* clades, respectively. However, because of a limited number of strains, further analysis of this potential species will need to be postponed until a sufficient amount of material obtained.

### Taxonomy

Based on the phylogenetic signals of the ITS and *tub2* and morphological characteristics, all strains analysed in the current study were assigned to six different groups (A–F). They represent one known species, *O.ips* (Rumbold 1931, Upadhyay 1981, Benade et al. 1995, Rane and Tattar 1987, Suh et al. 2013, Zhao et al. 2013) and two uncertain species (Gra.cf.rectangulosporium and O.cf.deltoideosporum) and the three species are hereby described as new species.

#### Sporothrix zhejiangensis

Taxon classificationFungiOphiostomatalesOphiostomataceae

Wang & Lu sp. nov.

MB825556

Figure 2

##### Etymology.

The epithet reflects Zhejiang Province in China where the species was first collected.

##### Type.

**CHINA**, Zhejiang, Yuyao City, from *Monochamusalternatus* gallery in *Pinusmassoniana* infested by numerous PWN, November 2012, collected by Q Lu and YY Lun, culture ex-holotype MUCL 55183 = CFCC52165 = CXY1614.

##### Description.

Sexual morph perithecial: Perithecia occasional on 2% MEA, emerging from the superficial mycelium or partly iμmersed, with a globose base, (75–)80–108(–120) μm in diameter, with some basal hyphal ornamentation, black; extending progressively into a straight, brown to black neck, (127–)156–550(–631) μm long, (26–)32–58.5(–65) μm wide at the base, (7–)7.5–10.7(–12) μm wide at the apex; ending in a crown of hyaline, (6–)9–19.5(–24) μm long ostiolar hyphae; ascospores reniform in side view, without sheath, aseptate, hyaline, (2–)2.2–3.4(–4) × (0.6–)0.74–2(–2.5) μm.

Asexual morph: pesotum-like and sporothrix-like.

Pesotum-like: Conidiophores macronematous, synnematous, abundant in 2% MEA. Synnemata occurring singly, enlarging towards both the apex and the base, dark brown at base, becoming paler toward the apex, (100–)120–260(–290) μm long including the conidiogenous apparatus, (56–)63–145(–158) μm wide at base, rhizoids present; conidiogenous cells (7–)9.5–29(–45.5) × 1–2(–1.7) μm; conidia hyaline, aseptate, single-celled, smooth, cylindrical or obovoid, (2–)2.5–4.8(–6) × (0.5–)0.8–2.1(–2.6) μm.

Sporothrix-like: Conidiophores micronematous, single on aerial mycelia, unbranched, (4.5–)9.6–31.5(–51.5) × (1.0–)1.5–2(–2.4) μm; conidia hyaline, smooth, aseptate, ellipsoid to ovoid, (2.5–)3–4.8(–5) × (0.7–)1–2.1(–2.5) μm.

**Figure 2.**
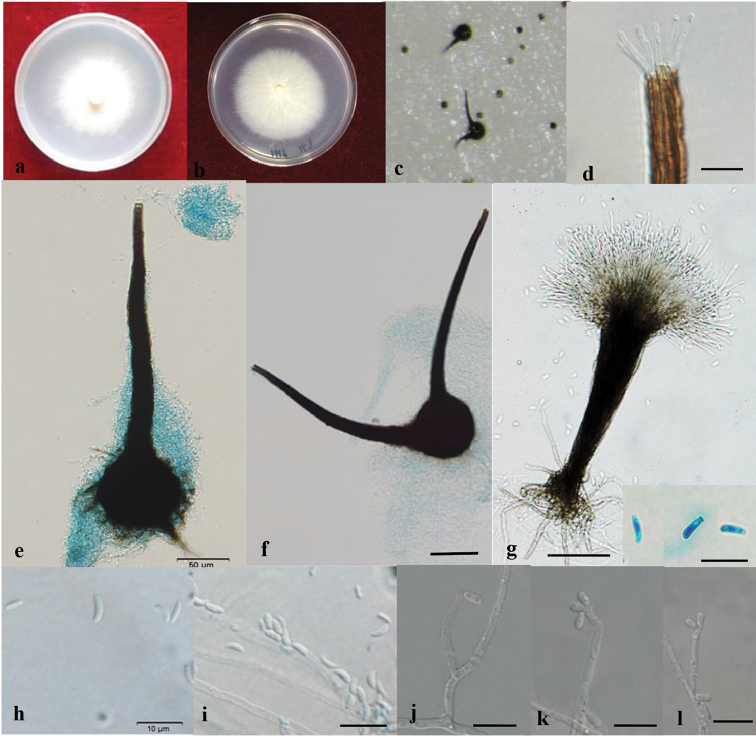
Light micrographs of *Sporothrixzhejiangensis*. **a–c** Growth on 2% MEA and 2% PDA, 2 weeks after inoculation **d** Occasionally observed ostiolar hyphae (scale bar, 20 μm) **e–f** Perithecium (scale bar, 20 μm) **g** Pesotum-like anamorph, rhizoid, conidiophores, conidiogenous apparatus (scale bar, 20 μm), and conidia (bottom right corner) (scale bar, 10 μm) **h, i** Reniform ascospores without sheaths (scale bar, 10 μm) **j–l**Sporothrix-like anamorph, conidiophores, and conidia (scale bar, 10 μm).

##### Culture characteristics.

Colonies on 2% MEA medium are white, with colony edge thinning radially. Hyphae are superficial on agar. Diameter reaches 50 μm in the dark after 8 d at 25 °C, able to grow at 5 °C and 40 °C, with the optimal growth temperature of 30 °C. Growth characteristics on PDA medium are similar.

##### Habitat and distribution.

Galleries of *Monochamusalternatus* in *Pinusmassoniana* infested by PWN; known hitherto from Zhejiang Province, China.

##### Additional specimens examined.

CHINA, Zhejiang, Yuyao City, from *Monochamusalternatus* galleries in *Pinusmassoniana* infested by PWN, November 2012, collected by Q Lu and YY Lun, MUCL 55181 = CFCC 52167 = CXY1612, MUCL 55182 = CFCC 52164 = CXY1613, MUCL 55184 = CFCC 52166 = CXY1615.

##### Note.

*Sporothrixzhejiangensis* is characterised by a sexual and two asexual forms (pesotum-like and sporothrix-like). It is phylogenetically related to *S.nebulare*, *S.eucalyptigena* and *S.epigloea* (Figure 1). *Sporothrixzhejiangensis* differs from *S.nebulare* in both ascomatal and conidial features. The perithecial neck of *S.nebulare* is shorter than that of *S.zhejiangensis*, respectively (140–)169–293(–365) μm and (127–)156–550(–631) μm. The conidia of *S.nebulare* also are smaller than those of *S.zhejiangensis*, mostly respectively 2.9–3.7 × 1.1–1.3 μm and 3–4.8 × 1–2.1 μm (Romón et al. 1900).

*Sporothrixeucalyptigena* and *S.epigloea* produce perithecia and ascospores similar to those of *S.zhejiangensis* (Crous et al. 2015, Upadhyay 1981). However, *S.eucalyptigena* has a slightly wider neck than *S.zhejiangensis* (20–35 *vs.* 9–19.5 μm) and longer ostiolar hyphae. Furthermore, *S.eucalyptigena* and *S.epigloea* only produce a sporothrix-like asexual state and their conidia differ from those of *S.zhejiangensis* either in size or in shape. *Sporothrixeucalyptigena* has drop-shaped (lacrymoid) conidia, differing from the ellipsoid to ovoid conidia in *S.zhejiangensis*. Conidia of *S.epigloea* are larger than those of *S.zhejiangensis* (2.5–9 × 1–3.5 *vs.* 3–4.8 × 1–2.1 μm) (Crous et al. 2015). Another conspicuous difference between *S.zhejiangensis* and *S.eucalyptigena* is the growth rate; the former grows much faster than the latter (50 μm in 8 d *vs.* 50 μm in 30 d at 25 °C) (Upadhyay 1981).

*Sporothrixzhejiangensis* is also closely related to *S.bragantina* and *S.thermara* (Figure 1) (Pfenning and Oberwinkler 1993, de Beer et al. 2016). These three species display the same optimal growth temperature (30 °C) and a similar conidial shape (ellipsoid to obovoid) of their sporothrix-like morph. However, the perithecial base of *S.bragantina* is larger than that of *S.zhejiangensis* [globose base: 130–220 μm *vs.* (75–)80–108(–120) μm and the neck also is longer, 700–1200 μm *vs.* (127–)156–550(–631) μm]. The sporothrix-like conidia of *S.bragantina* also are larger than those of *S.zhejiangensis* (4–6 × 2–2.5 μm *vs.* 3–4.8 × 1–2.1 μm). *Sporothrixthermara*, hitherto, has no known sexual state. It only known by sporothrix-like state; conidia of *S.thermara* are larger than those of *S.zhejiangensis* (4–6 × 2–3 μm *vs.* 3–4.8 × 1–2.1 μm).

#### Ophiostoma album

Taxon classificationFungiOphiostomatalesOphiostomataceae

Wang & Lu sp. nov.

MB825557

Figure 3

##### Etymology.

The epithet reflects the white colour of the colonies.

##### Type.

**CHINA**, Zhejiang, Yuyao City, from *Monochamusalternatus* gallery of *Pinusmassoniana* infested by numerous PWN, November 2012, collected by Q Lu and YY Lun, culture ex-holotype MUCL 55189 = CFCC 52168 = CXY1622.

##### Description.

Sexual form: Unknown. Asexual form: Hyalorhinocladiella-like. Conidiogenous cells micronematous, (4.2–)9.5–16.5(–20.5) × (0.5–)1–2(–2.5) μm; conidia hyaline, single-celled, aseptate, clavate or fusiform obovoid with pointed bases and (occasionally) rounded apices, slightly curved at the base (4–)4.2–14.5(–18) × (0.5–)1–2(–2.3) μm.

**Figure 3.**
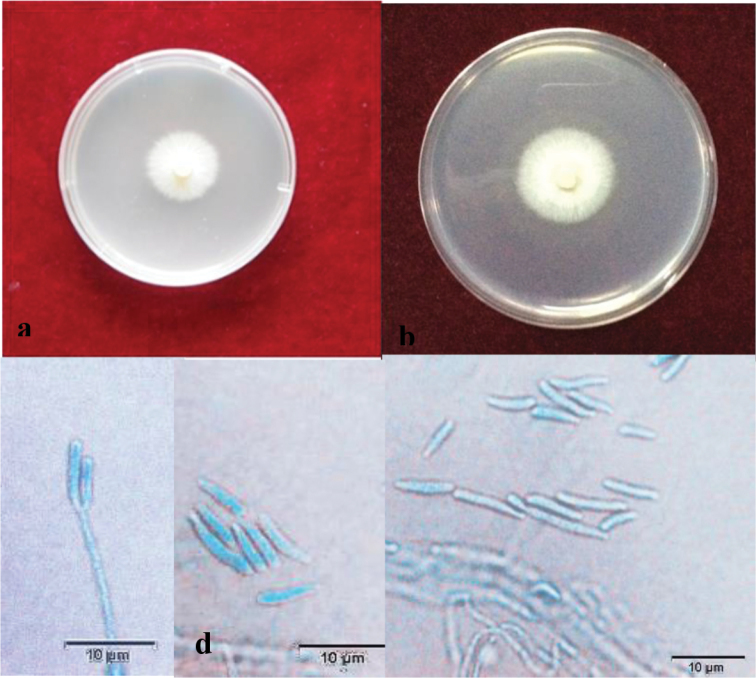
Light micrographs of *Ophiostomaalbum*. **a, b** Growth on 2% MEA and 2% PDA, 2 weeks after inoculation **c–e**Hyalorhinocladiella-like anamorph, conidiophores, and conidia (scale bar, 10 mm).

##### Culture characteristics.

Colonies on 2% MEA white, with the mycelium edge thinning radially; Hyphae are superficial on agar, sporulation weak. Colonies slowly growing, reaching 18.5 μm in diameter at 8 d at 25 °C, able to grow at 40 °C but not at 5 °C, with the optimal growth temperature of 35 °C. Growth characteristics on PDA culture medium are similar but the growth rate is slower than on MEA.

##### Habitat and distribution.

Galleries of *Monochamusalternatus* in *Pinusmassoniana*, infested by PWN, in Zhejiang Province, China.

##### Additional specimens examined.

CHINA, Zhejiang, Yuyao City, from *Monochamusalternatus* galleries of *Pinusmassoniana* infested by numerous PWN, November 2012, collected by Q Lu and YY Lun, MUCL 55190 = CFCC 52169 = CXY1642, CXY1643 = CFCC 52170.

##### Note.

*Ophiostomaalbum* only known in its asexual hyalorhinocladiella-like form. According to both ITS and *tub2* based phylogenetic analysis, it is closely related to *O.kryptum* and *O.olgensis* in the *O.minus* complex (Figure 1). *Ophiostomaalbum* is easily distinguished from *O.olgensis* and *O.kryptum* based on their reproduction structure. *Ophiostomaalbum* only produces a hyalorhinocladiella-like asexual form *in vitro*, whereas the two other species produce both a sexual and asexual forms *in vitro* (Jacobs and Kirisits 2003, Wang et al. 2016). The conidial size and shape of the three species are obviously different. *Ophiostomaalbum* produces clavate or fusiform to obovoid and sometimes, slightly curved conidia; these are obovoid with pointed bases in both *O.olgensis* and *O.kryptum*. Furthermore, the conidia of *O.album* are much larger, 4.2–14.5 × 1.0–1.9 μm *vs.* 1.5–7 × 1.5–5 μm in the two other species.

#### Ophiostoma massoniana

Taxon classificationFungiOphiostomatalesOphiostomataceae

Wang & Lu sp. nov.

MB825558

Figure 4

##### Etymology.

The epithet reflects the host tree, *Pinusmassoniana*.

##### Type.

**CHINA**, Zhejiang Province, Fuyang City, from *Monochamusalternatus* gallery in *Pinusmassoniana* infested by numerous PWN, November 2012, collected by Q Lu and YY Lun, culture ex-holotype, MUCL 55179 = CFCC 51648 = CXY1610.

##### Description.

Sexual form: Unknown. Asexual form: Hyalorhinocladiella-like. Conidiophores abundant, single, borne on aerial hyphae, (3.3–)10.5–27.5(–42.5) × (0.7–)1.3–2.0(–2.7) μm; conidia hyaline, single-celled, aseptate, obovoid or globose with pointed bases and rounded apices, (2–)2.2–3.9(–5) × (0.5–)0.7–1.7(–2) μm.

**Figure 4.**
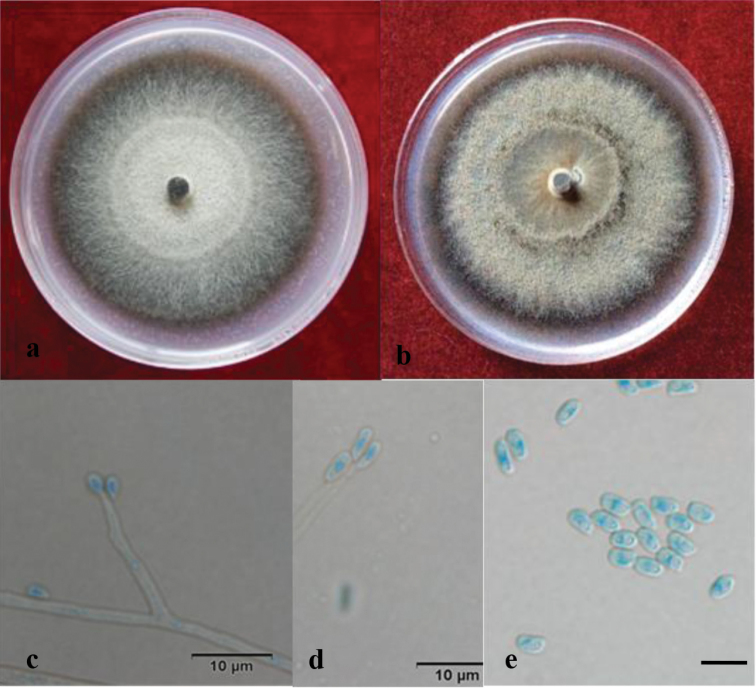
Light micrographs of *Ophiostomamassoniana*. **a, b** Growth on 2% MEA and 2% PDA, 2 weeks after inoculation **c–e**Hyalorhinocladiella-like anamorph, conidiophores, conidia (scale bar, 10 μm).

##### Culture characteristics.

Colonies on 2% MEA brown, the marginal hyphae sparse and radiating; some white mycelium produced early during growth that becomes black after 3–5 d. Colonies slowly growing, reaching 37.5 μm in diameter over 8 d at 25 °C, able to grow at 5 °C and 40 °C, with an optimal growth temperature of 30 °C; sporulation weak. On PDA culture medium, the colonies are dark brown; the mycelium is white, long and dense, with a daily growth of 4 μm at 25 °C.

##### Habitat and distribution.

Galleries of *Monochamusalternatus* in *Pinusmassoniana* infested by PWN, in Zhejiang Province, China.

##### Additional specimens examined.

CHINA, Zhejiang Province, Yuyao City, from *Monochamusalternatus* galleries in *Pinusmassoniana* infested by numerous PWN, November 2012, collected by Q Lu and YY Lun, MUCL 55180 = CFCC 51649 = CXY1611.

##### Note.

*Ophiostomamassoniana*, only known by its asexual, hyalorhinocladiella-like state, does not cluster in any of the 10 species complexes defined by de Beer and Wingfield (2013) in *Ophiostoma* s. l. According to the ITS and *tub2* phylogenetic analysis, the species is related to *O.saponiodorum* and *O.pallidulum* (Figure 1). *Ophiostomapallidulum* also only produces asexual hyalorhinocladiella-like morphs *in vitro*, whereas *O.saponiodorum* produces a sexual and two asexual morphs (pesotum-like and hyalorhinocladiella-like). In addition, *O.massoniana* differs from *O.saponiodorum* in producing smaller conidia [(2–)2.2–3.9(–5) × (0.5–)0.7–1.7(–2) μm vs. (3–)4–6(–7) × 1–1.5(–2) μm] (Linnakoski et al. 2010). Further, the colour of *O.massoniana* colonies is different from that of the other two species. Namely, *O.massoniana* forms brown to dark brown colonies, while the other two species form pale colonies (Linnakoski et al. 2010).

## Discussion

In the current study, six ophiostomatoid species were found associated with pines infected by *M.alternatus* and PWN in the eastern provinces of Shandong and Zhejiang in China: *O.ips*, the newly described *S.zhejiangensis*, *O.album*, *O.massoniana* and two species whose identities are uncertain; O.cf.deltoideosporum and Gra.cf.rectangulosporium. *Ophiostomaips* was the most frequently isolated species, accounting for over 90% of all Ophiostomatales strains.

*Ophiostomaips* was originally reported in association with bark beetles infecting pines in south-eastern North America (Rumbold 1931). It has been since reported in Central and South America (Mexico and Chile), Europe (Austria and Sweden), Asia (China, Japan and Korea), Africa (South Africa) and Australasia (New Zealand) (Rumbold 1931, Benade et al. 1995, Rane and Tattar 1987, Zhou et al. 2002; Lu et al. 2009, Suh et al. 2013, Zhao et al. 2013; 2014). Furthermore, *O.ips* is a ubiquitous sapstain fungus associated with PWN and *Monochamus* spp. (Zhao et al. 2014).

In China, *O.ips* was reportedly associated with *P.massoniana* infected by PWN (Zhao 1992, Zhao et al. 2006, 3013) and with *P.tabuliformis* infected by *Dendroctonusvalens* (Lu et al. 2009), two invasive pests of the local conifer ecosystems. Zhao et al. (2013) reported *O.ips* an isolation frequency of 37% in three ophiostomatoid fungal communities associated with PWN, much lower than that reported in the current study.

*Ophiostomaips* appears to have travelled long-distances in wood materials presumably originating from North America (Zhou et al. 2007). The cited study did not consider any Asian population, however. Nevertheless, the high population density of *O.ips* in China suggests either indigenous origin or effective adaption after the invasion to local pine forests, with a long evolution history. To verify this hypothesis, it will be necessary to analyse the dispersal routes of PWN populations in different areas globally and of the fungus–including Asian populations.

Members of *Sporothrix* are reportedly associated with a wide range of habitats (De Hoog 1974, Kwon-Chung and Bennet 1992, Roets et al. 2006, Zhou et al. 2006, Madrid et al. 2009), e.g. wood (Aghayeva et al. 2004), human (de Beer et al. 2016) and the soil (De Meyer et al. 2008). The genus is characterised by reniform ascospores without a mucilaginous sheath and sporothrix- and pesotum-like asexual states (Linnakoski et al. 2010, de Beer et al. 2013). Genetically, the species of the *Sporothrix* lineages lack the intron 4 but have intron 5 in the BT gene (Zipfel et al. 2006).

*Sporothrixzhejiangensis* forms an independent lineage according to both ITS and *tub2* based on phylogenetic inferences. It is closely related to *S.nebulare*, *S.eucalyptigena*, *S.epigloea*, *S.bragantina* and *S.thermara* (Madrid et al. 2010, Romón et al. 1900, Crous et al. 2015, de Beer et al. 2016, Van der Linde et al. 2016) (Figure 1). *Sporothrixnebulare* was first described after isolation from *Hylastesattenuatus* infesting *P.radiata* in Spain (Romón et al. 1900). *Sporothrixeucalyptigena* was recently isolated from *Eucalyptusmarginata* (Myrtaceae) in Western Australia (Crous et al. 2015). *Sporothrixepigloea* was isolated from *Tremellafuciformis* in Argentina (Upadhyay 1981). *S.bragantina* was isolated from the rhizosphere soil in Brazil (Pfenning and Oberwinkler 1993) and *S.thermara* from *Cyrtogeniusafricus* galleries in diseased *Euphorbiaingens* trees in South Africa (Van der Linde et al. 2016). Hence, *S.zhejiangensis* and these five species differ with respect to their (known) hosts and geographic distributions.

Although *S.zhejiangensis* is unrelated to *S.fusiforis*, *S.lunata* and *S.stenoceras* (Figure 1), these strains exhibit a similar sexual state (Hsiau 1996, Yamaoka et al. 2000, Aghayeva et al. 2004, Zhou et al. 2004). For instance, they all develop one to two perithecial necks emerging from the globular base; occasionally, abnormal specimens of *O.stenoceras* develop up to five necks *in vitro* (Yamaoka et al. 2000).

In the current study, *S.zhejiangensis* was notably different from *Sporothrix* sp. 1 and *Sporothrix* sp. 2 (Zhao et al. 2013) with regard to colony characteristics (*S.zhejiangensis* has a white and radially thinning edge; *Sporothrix* sp. 1: dark, superficial mycelium; *Sporothrix* sp. 2: white, radially dense mycelium). Consequently, the role of *S.zhejiangensis* in PWN needs further research and analysis, ruling out the possibility that the species had been already discovered and its ecological role partially studied.

According to ITS phylogeny analysis, *Ophiostomaalbum* is related to *O.olgensis* (Wang et al. 2016) in a single but weakly supported clade (Figure 1). This clade nests within the *O.minus* complex, in which it is closely related to *O.kryptum* (Jacobs and Kirisits 2003). The *tub2* dataset confirmed that *O.album* and *O.olgensis* formed two clades.

The *O.minus* complex currently includes *O.minus*, *O.pseudotsugae*, *O.allantosporum*, *O.kryptum* and *O.olgensis* (Jacobs and Kirisits 2003, Gorton et al. 2004, de Beer and Wingfield 2013, Wang et al. 2016). The *tub2* gene of the *O.minus* complex members includes intron 4 but lacks intron 5 (Gorton et al. 2004). *Ophiostomaalbum* is phylogenetically closely related to *O.olgensis* and *O.kryptum*. Both *O.olgensis* and *O.kryptum* inhabit *Larix* spp. (Jacobs and Kirisits 2003; Wang et al. 2016), whereas *O.album* inhabits *P.massoniana*. Both *O.olgensis* and *O.album* occur in China, whereas *O.kryptum* is found in central Europe. Moreover, the three species are associated with different vectors (Jacobs and Kirisits 2003, Wang et al. 2016).

According to both ITS and *tub2* phylogenetic trees, *O.massoniana* forms a separated well-supported clade (Figure 1). It groups with *O.pallidulum* and *O.saponiodorum* (Figure 1), which has been isolated from *Pinussylvestris* in Finland and *Piceaabies* in Russia in association with various bark beetles (Linnakoski et al. 2010). The three species produce a hyalorhinocladiella-like asexual form (Linnakoski et al. 2010; de Beer et al. 2013) and their *tub2* genes lack intron 4 but contain intron 5 (Zipfel et al. 2006).

## Conclusions

In the current study, a relatively large number of ophiostomatoid fungal species associated with *B.xylophilus* and *M.alternatus* in Shandong and Zhejiang Provinces in China was identified. Three novel species, *O.album*, *O.massoniana* and *S.zhejiangensis* were discovered and described. Fourteen additional provinces in China are currently also listed as PWN epidemic areas (State Forestry Administration of the People’s Republic of China 2018). Hence, additional ophiostomatoid fungi associated with *B.xylophilus* and *M.alternatus* should be discovered and described. Future in-depth studies of the biodiversity, biogeography and ecology of fungi associated with pine wilt disease will contribute to the understanding of disease mechanisms and provide information on effective management methods to alleviate the subsequent plant losses.

## Supplementary Material

XML Treatment for Sporothrix zhejiangensis

XML Treatment for Ophiostoma album

XML Treatment for Ophiostoma massoniana

## Supplementary materials

Supplementary material 1 Figure S1. Phylogram of fungal associates of pine infected by PWN and *Monochamusalternatus* in China Data type: phylogenetic data Explanation note: The phylogram was generated after MP analysis of partial *tub2* sequences. *O.ips* sequences obtained in the current study are designated in bold type. MP bootstrap value and BI values are indicated at the branch nodes; values below 70% are indicated by asterisk (*). This is an open access article distributed under the terms of the Creative Commons Attribution License (CC BY 4.0), which permits unrestricted use, distribution, and reproduction in any medium, provided the original author and source are credited.HuiMin Wang, YingYing Lun, Quan Lu, HuiXiang Liu, Cony Decock, XingYao Zhang

Supplementary material 2 Figure S2. Phylograms of fungal associates of pine infected by PWN and *Monochamusalternatus* in China Data type: phylogenetic data Explanation note: The phylograms were generated after MP analysis of the ITS1–5.8S–ITS2 rDNA and partial *tub2* sequences. Novel sequences obtained in the current study are indicated in bold type. MP bootstrap values (10,000 replicates) and ML bootstrap support values (1000 replicates) (normal type) above 70% are indicated at the nodes. Values below 70% are indicated by asterisk (*). Posterior probabilities (above 90%) obtained from BI are indicated by bold lines at the relevant branching points. Scale bar, total nucleotide differences between taxa; ML, maximum likelihood; MP, maximum parsimony; BI, Bayesian inference. This is an open access article distributed under the terms of the Creative Commons Attribution License (CC BY 4.0), which permits unrestricted use, distribution, and reproduction in any medium, provided the original author and source are credited.HuiMin Wang, YingYing Lun, Quan Lu, HuiXiang Liu, Cony Decock, XingYao Zhang

Supplementary material 3 Figure S3. Three ML phylogenetic threes based on tub2 after excluding introns Data type: phylogenetic data This is an open access article distributed under the terms of the Creative Commons Attribution License (CC BY 4.0), which permits unrestricted use, distribution, and reproduction in any medium, provided the original author and source are credited.HuiMin Wang, YingYing Lun, Quan Lu, HuiXiang Liu, Cony Decock, XingYao Zhang
