# Supplementary material for: Ophiostomatoid fungi associated with pines infected by Bursaphelenchusxylophilus and Monochamusalternatus in China, including three new species
Source: MycoKeys. 2018 Sep 4;(39):1–27. doi: 10.3897/mycokeys.39.27014 (PMC6182259; doi:10.3897/mycokeys.39.27014)
Supplement: Supplementary material 1 — Figure S1. Phylogram of fungal associates of pine infected by PWN and Monochamusalternatus in China [file mycokeys-39-001-s001.docx]

5 changes

**CXY1628**

**CXY1631**

**CXY1635**

**CXY1638**

*O*.*ips* AY194949

*O.ips* AY194951

*O.ips* KC588951

*O.pulvinisporum* DQ296100

*O.pulvinisporum* EU977487

*O.bicolor* DQ268636

*O.bicolor* HM031562

*O.bicolor* HM031560

*O.novo ulmi* EU977486

*O.quercus* AY789157

*O.montium* AY194957

**CXY1639**

82\0.96

100\1.00

*O.piceae* FJ430514

100\1.00

100\1.00

85\0,87

82\0,79

*O.canum* GQ423509

*O.floccosum* AY789142

77\0.79

*\*

*\0,81

**Appendix: Figure 1.** Phylogram of fungal associates of pine infected by PWN and *M*. *alternatus* in China. The phylogram was generated after MP analysis of partial *tub2* sequences. *O*. *ips* sequences obtained in the current study are designated in bold type. MP bootstrap value and BI values are indicated at the branch nodes; values below 70% are indicated by asterisk (_*_).
