# Supplementary material for: Ophiostomatoid fungi associated with pines infected by Bursaphelenchusxylophilus and Monochamusalternatus in China, including three new species
Source: MycoKeys. 2018 Sep 4;(39):1–27. doi: 10.3897/mycokeys.39.27014 (PMC6182259; doi:10.3897/mycokeys.39.27014)
Supplement: Supplementary material 2 — Figure S2. Phylograms of fungal associates of pine infected by PWN and Monochamusalternatus in China [file mycokeys-39-001-s002.pdf]

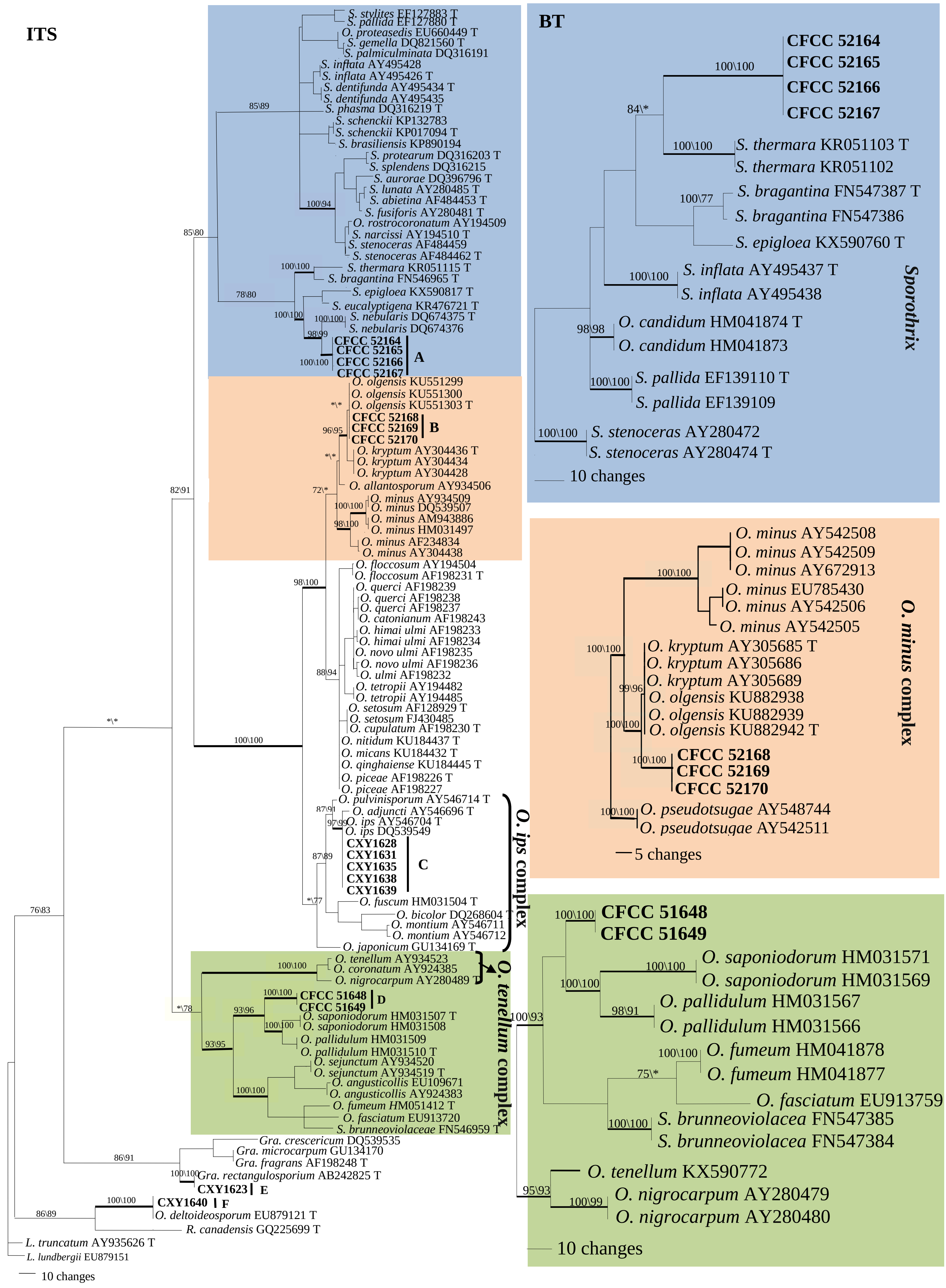
ITS
S. stylites EF127883 T
S. pallida EF127880 T
O. proteasedis EU660449 T
S. gemella DQ821560 T
S. palmiculminata DQ316191
S. inflata AY495428
S. inflata AY495426 T
S. dentifunda AY495434 T
S. dentifunda AY495435
85\89
S. phasma DQ316219 T
S. schenckii KP132783
S. schenckii KP017094 T
S. brasiliensis KP890194
S. protearum DQ316203 T
S. splendens DQ316215
S. aurorae DQ396796 T
S. lunata AY280485 T
100\94
S. abietina AF484453 T
S. fusiforis AY280481 T
O. rostrocoronatum AY194509
S. narcissi AY194510 T
85\80
S. stenoceras AF484459
S. stenoceras AF484462 T
100\100
S. thermara KR051115 T
S. bragantina FN546965 T
78\80
S. epigloea KX590817 T
S. eucalyptigena KR476721 T
100\100
100\100
S. nebularis DQ674375 T
S. nebularis DQ674376
98\99
CFCC 52164
CFCC 52165
100\100
CFCC 52166
A
CFCC 52167
O. olgensis KU551299
O. olgensis KU551300
**
O. olgensis KU551303 T
CFCC 52168
96\95
CFCC 52169
B
CFCC 52170
O. kryptum AY304436 T
**
O. kryptum AY304434
O. kryptum AY304428
82\91
72*
O. allantosporum AY934506
O. minus AY934509
100\100
O. minus DQ539507
O. minus AM943886
98\100
O. minus HM031497
O. minus AF234834
O. minus AY304438
O. floccosum AY194504
O. floccosum AF198231 T
98\100
O. querci AF198239
O. querci AF198238
O. querci AF198237
O. catonianum AF198243
O. himai ulmi AF198233
O. himai ulmi AF198234
O. novo ulmi AF198235
88\94
O. novo ulmi AF198236
O. ulmi AF198232
O. tetropii AY194482
O. tetropii AY194485
**
O. setosum AF128929 T
O. setosum FJ430485
O. cupulatum AF198230 T
100\100
O. nitidum KU184437 T
O. micans KU184432 T
O. qinghaiense KU184445 T
O. piceae AF198226 T
O. piceae AF198227
O. pulvinisporum AY546714 T
87\91
O. adjuncti AY546696 T
97\99
O. ips AY546704 T
O. ips DQ539549
CXY1628
CXY1631
87\89
CXY1635
C
CXY1638
CXY1639
O. ips complex
*\77
O. fuscum HM031504 T
O. bicolor DQ268604 T
76\83
O. montium AY546711
O. montium AY546712
O. japonicum GU134169 T
O. tenellum AY934523
100\100
O. coronatum AY924385
O. nigrocarpum AY280489 T
100\100
CFCC 51648
D
CFCC 51649
*\78
93\96
O. saponiodorum HM031507 T
100\100
O. saponiodorum HM031508
O. pallidulum HM031509
93\95
O. pallidulum HM031510 T
O. tenellum complex
O. sejunctum AY934520
O. sejunctum AY934519 T
O. angusticollis EU109671
100\100
O. angusticollis AY924383
O. fumeum HM051412 T
O. fasciatum EU913720
S. brunneoviolaceae FN546959 T
Gra. crescericum DQ539535
86\91
Gra. microcarpum GU134170
Gra. fragrans AF198248 T
100\100
Gra. rectangulosporium AB242825 T
CXY1623
E
100\100
CXY1640
F
86\89
O. deltoideosporum EU879121 T
R. canadensis GQ225699 T
L. truncatum AY935626 T
L. lundbergii EU879151
10 changes
BT
100\100
CFCC 52164
CFCC 52165
CFCC 52166
84*
CFCC 52167
100\100
S. thermara KR051103 T
S. thermara KR051102
100\77
S. bragantina FN547387 T
S. bragantina FN547386
S. epigloea KX590760 T
100\100
S. inflata AY495437 T
Sporothrix
S. inflata AY495438
98\98
O. candidum HM041874 T
O. candidum HM041873
100\100
S. pallida EF139110 T
S. pallida EF139109
100\100
S. stenoceras AY280472
S. stenoceras AY280474 T
10 changes
O. minus AY542508
O. minus AY542509
100\100
O. minus AY672913
O. minus EU785430
O. minus AY542506
O. minus AY542505
100\100
O. kryptum AY305685 T
O. kryptum AY305686
O. kryptum AY305689
99\96
O. olgensis KU882938
O. minus complex
O. olgensis KU882939
100\100
O. olgensis KU882942 T
100\100
CFCC 52168
CFCC 52169
CFCC 52170
100\100
O. pseudotsugae AY548744
O. pseudotsugae AY542511
5 changes
100\100
CFCC 51648
CFCC 51649
O. saponiodorum HM031571
100\100
100\100
O. saponiodorum HM031569
O. pallidulum HM031567
98\91
100\93
O. pallidulum HM031566
100\100
O. fumeum HM041878
75*
O. fumeum HM041877
O. fasciatum EU913759
100\100
S. brunneoviolacea FN547385
S. brunneoviolacea FN547384
O. tenellum KX590772
95\93
O. nigrocarpum AY280479
100\99
O. nigrocarpum AY280480
10 changes

Figure 1. Phylograms of fungal associates of pine infected by PWN and *Monochamus alternatus* in China. The phylograms were generated after MP analysis of the ITS1–5.8S–ITS2 rDNA and partial *tub2* sequences. Novel sequences obtained in the current study are indicated in bold type. MP bootstrap values (10,000 replicates) and ML bootstrap support values (1000 replicates) (normal type) above 70% are indicated at the nodes. Values below 70% are indicated by asterisk (*). Posterior probabilities (above 90%) obtained from BI are indicated by bold lines at the relevant branching points. Scale bar, total nucleotide differences between taxa; ML, maximum likelihood; MP, maximum parsimony; BI, Bayesian inference.
