# Supplementary figures and images for: Ophiostomatoid fungi associated with pines infected by Bursaphelenchusxylophilus and Monochamusalternatus in China, including three new species
Source: MycoKeys. 2018 Sep 4;(39):1–27. doi: 10.3897/mycokeys.39.27014 (PMC6182259; doi:10.3897/mycokeys.39.27014)

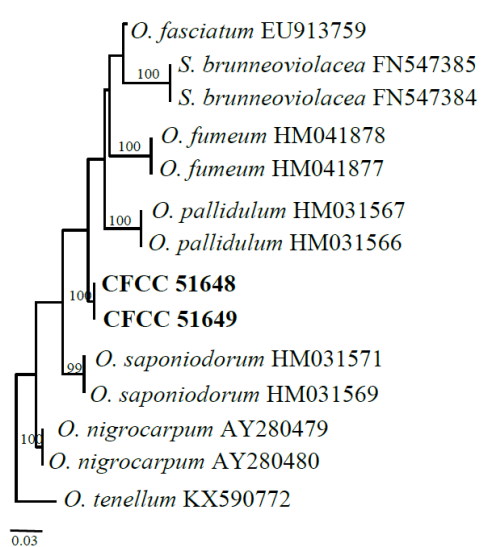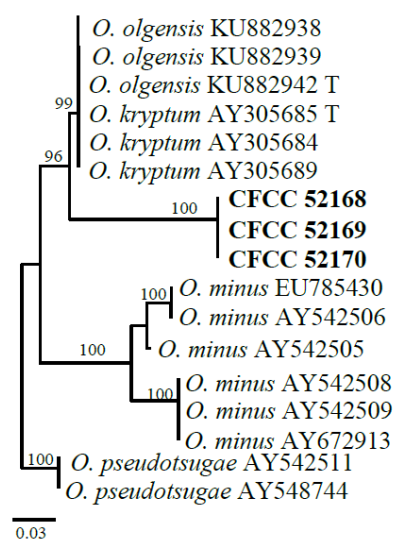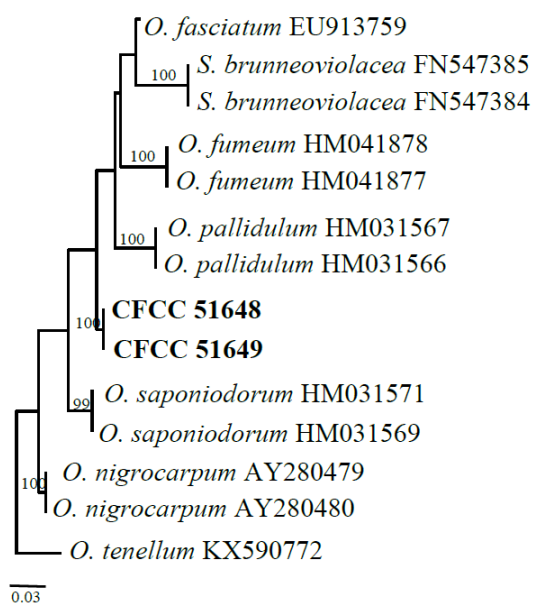

Appendix Fig 3: Three ML phylogenetic trees based on *tub2* after excluding introns.

Supplement: Supplementary material 3 — Figure S3. Three ML phylogenetic threes based on tub2 after excluding introns [file mycokeys-39-001-s003.pdf]
